# Toxin-neutralizing Abs are associated with improved T cell function following recovery from *Staphylococcus aureus* infection

**DOI:** 10.1172/jci.insight.173526

**Published:** 2024-01-18

**Authors:** Maureen Kleinhenz, Zhaotao Li, Usha Chidella, Walissa Picard, Amber Wolfe, Jill Popelka, Robin Alexander, Christopher P. Montgomery

**Affiliations:** 1 Center for Microbial Pathogenesis, Abigail Wexner Research Institute;; 2 Division of Critical Care Medicine; and; 3 Biostatistics Resource, Nationwide Children’s Hospital, Columbus, Ohio, USA.; 4 Department of Pediatrics, The Ohio State University College of Medicine, Columbus, Ohio, USA.

**Keywords:** Immunology, Infectious disease, Adaptive immunity, Bacterial infections, T cells

## Abstract

**BACKGROUND:**

T cell responses are impaired in *Staphylococcus aureus*–infected children, highlighting a potential mechanism of immune evasion. This study tested the hypotheses that toxin-specific antibodies protect immune cells from bacterial killing and are associated with improved T cell function following infection.

**METHODS:**

*S*. *aureus*–infected and healthy children (*N* = 33 each) were prospectively enrolled. During acute infection and convalescence, we quantified toxin-specific IgG levels by ELISA, antibody function using a cell killing assay, and functional T cell responses by ELISPOT.

**RESULTS:**

There were no differences in toxin-specific IgG levels or ability to neutralize toxin-mediated immune cell killing between healthy and acutely infected children, but antibody levels and function increased following infection. Similarly, T cell function, which was impaired during acute infection, improved following infection. However, the response to infection was highly variable; up to half of children did not have improved antibody or T cell function. Serum from children with higher α-hemolysin–specific IgG levels more strongly protected immune cells against toxin-mediated killing. Importantly, children whose serum more strongly protected against toxin-mediated killing also had stronger immune responses to infection, characterized by more elicited antibodies and greater improvement in T cell function following infection.

**CONCLUSION:**

This study demonstrates that, despite T cell impairment during acute infection, *S*. *aureus* elicits toxin-neutralizing antibodies. Individual antibody responses and T cell recovery are variable. These findings also suggest that toxin-neutralizing antibodies protect antigen-presenting cells and T cells, thereby promoting immune recovery. Finally, failure to elicit toxin-neutralizing antibodies may identify children at risk for prolonged T cell suppression.

**FUNDING:**

NIH National Institute of Allergy and Infectious Diseases R01AI125489 and Nationwide Children’s Hospital.

## Abstract 2

<p><em>S. aureus</em> infection elicits&nbsp;heterogeneous antibody and T cell responses, and antibody-mediated protection of immune cells from toxin-mediated killing correlates&nbsp;with recovery of T cell impairment.</p>

## Graphical abstract

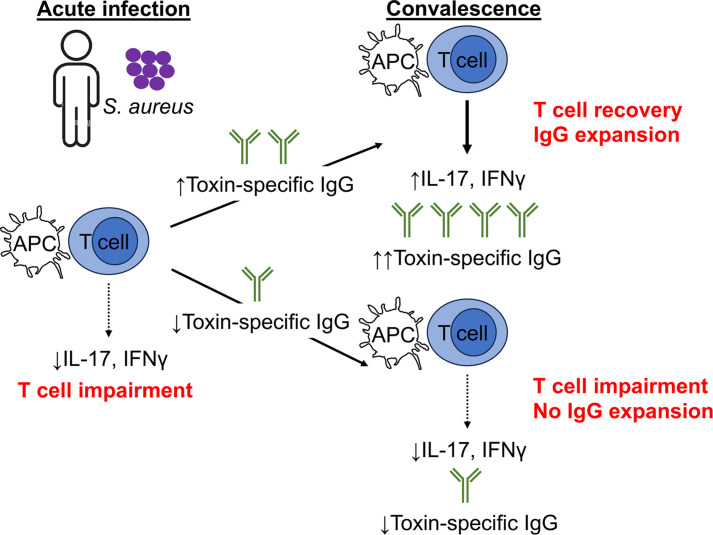

## Introduction

*Staphylococcus aureus* is a leading cause of infections in community and health care settings, resulting in substantial morbidity (> 700,000 hospitalizations/yr in the United States) and mortality (> 20,000 deaths/yr in the United States) (1–3). *S*. *aureus* is a pathobiont that asymptomatically colonizes mucosal surfaces, such as the nose, skin, and gastrointestinal tract, in up to 50% of individuals (4, 5) but also causes infections ranging from mild (e.g., skin and soft tissue infections, SSTIs) to severe (e.g., osteoarticular infections, pneumonia, endocarditis, bacteremia, and septic shock) (1). *S*. *aureus* is a leading cause of infection-related hospitalizations in children (3). Recurrent infections are common, occurring in up to 50% of otherwise-healthy children and adults within a year (6, 7). Coupled with the emergence of antibiotic-resistant *S*. *aureus* isolates, the burden of infection has led the field to prioritize the development of a vaccine to prevent these infections.

Unfortunately, every candidate vaccine has failed in clinical trials (8–11). Potential reasons for these failures include: (i) animal models that fail to recapitulate human infection (12–14), (ii) target populations that have primarily included adults at high risk of *S*. *aureus* infection (9–11), (iii) antigen selection, and (iv) vaccines that have targeted protective antibody. Underlying these challenges, the “natural” development of antistaphylococcal immunity, either in healthy individuals or during infection, remains poorly understood. Therefore, natural protective immunity against *S*. *aureus* has not been leveraged in vaccine design. As an example, the sole identified correlate of protection against recurrent infection is an increase in α-hemolysin (Hla) antibody levels, but the mechanism by which this may protect is not clear (6). While antigen-specific antibody levels have been well documented in children and adults (15–19), much less is understood regarding the development of T cells that drive immunologic memory. We previously reported that, despite high levels of antibodies against Hla and the leukotoxin subunits leukotoxin E (LukE) and Panton-Valentine leukocidin S (LukS-PV), children have markedly fewer *S*. *aureus*– and toxin-specific IFN-γ– and IL-17–secreting T cells during acute *S*. *aureus* infection, compared with healthy children (20). Therefore, evasion of T cell–mediated immunity may be a phenotype of *S*. *aureus* infection. However, it is unclear if impaired T cell responses predate acute infection, and therefore could contribute to susceptibility to infection, or if T cells are actively suppressed during infection.

In the current study, we sought to expand on our previous findings during acute infection by quantifying antibody levels and T cell responses in a cohort of children during acute infection and following recovery from infection. We found that toxin-specific antibody levels increased during convalescence in many, but not all, children and that Hla-specific antibody levels correlated with the ability of patient serum to protect immune cells from toxin-mediated killing. Similarly, impaired T cell responses improved following resolution of infection in many, but not all, children. This suggests that T cell impairment recovers following infection in a subset of children. Importantly, children with more toxin-neutralizing antibody had stronger immune responses to infection, characterized by stronger infection-elicited antibody responses and better recovery of T cell function. Together, these findings highlight the variability of infection-elicited immune responses and suggest a potential role for toxin-specific antibodies in protecting antigen-presenting cells and T cells and promoting recovery of T cell function.

## Results

### Characteristics of study participants.

Thirty-three hospitalized children with *S*. *aureus* infection were enrolled between September 2020 and December 2021 at Nationwide Children’s Hospital (Table 1). The median age of infected children was 10 years, 76% were male, and 82% were White. Infections were classified as invasive (52%) or noninvasive (48%); the most common sites of infection were blood (65% of invasive infections) and SSTI (100% of noninvasive infections). The majority of *S*. *aureus* isolates were methicillin susceptible for both invasive (70%) and noninvasive (56%) infections. Thirty-three healthy children (matched for age, race, and sex) were enrolled from May 2019 through January 2022. The median age of healthy controls was 7.9 years, 67% of healthy controls were male, and 76% were White. There were no significant differences between infected and healthy children with respect to age, biological sex, or race.

### Anti-–toxin IgG levels increase following infection in a subset of infected children.

**Figure 1:**
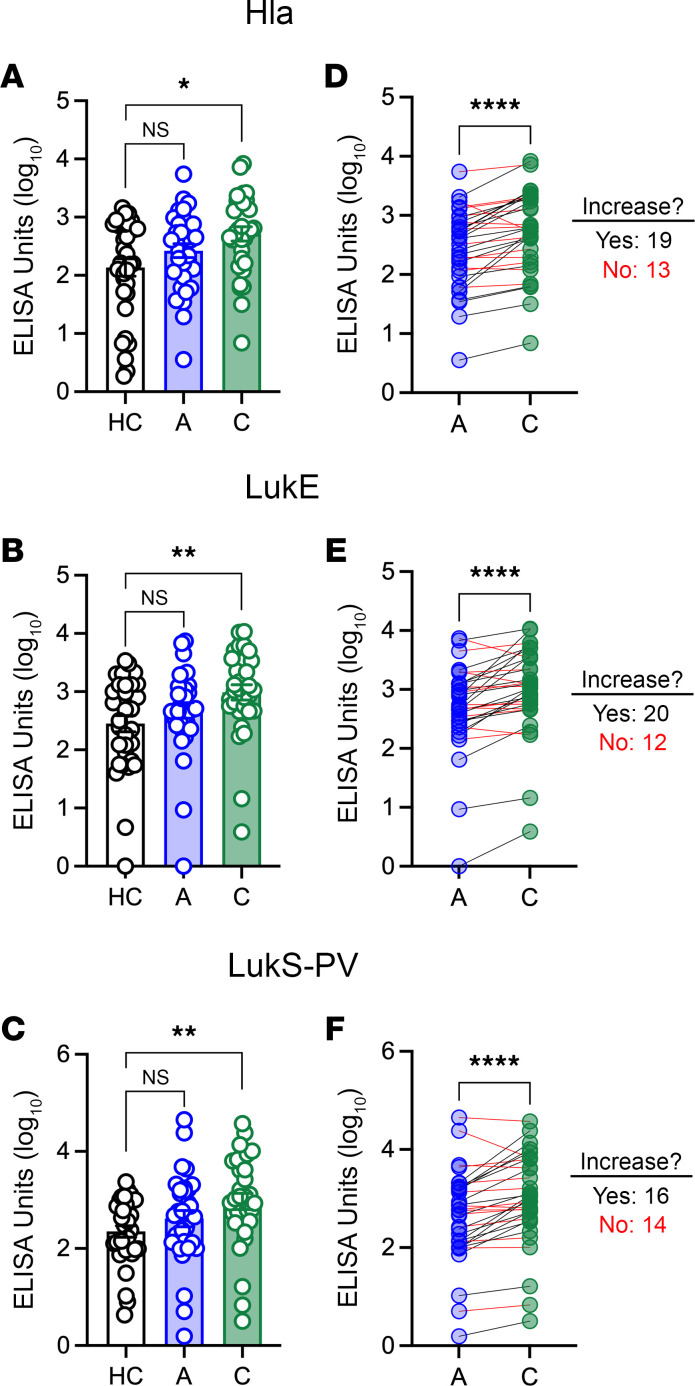
Anti-toxin IgG levels during acute infection and convalescence. (**A**–**C**) Levels of IgG against Hla (**A**), LukE (**B**), and LukS-PV (**C**) in healthy children (HC), during acute *S*. *aureus* infection (“A”), or during convalescence (“C”). (**D**–**F**) Change in IgG levels for individual study participants from acute infection to convalescence. Black lines indicate participants for whom IgG levels increased ≥ 1.5-fold; red lines indicate participants for whom IgG levels did not increase. Data are expressed as arbitrary ELISA units (log_10_) and presented as the median values with individual values superimposed on the plots. Log_10_-transformed values were compared by 1-way ANOVA with Kruskal-Wallis posttest (panels **A**–**C**) or Wilcoxon matched pairs signed rank test (panels **D**–**F**). *P < 0.05; **P < 0.01; ****P < 0.0001.

We quantified IgG levels against the staphylococcal toxins Hla, LukE, and LukS-PV by ELISA in 33 healthy and 32 infected children (1 infected child did not have a serum sample available). As we previously reported (20), there were no significant differences in IgG levels between children during acute *S*. *aureus* infection and healthy children, but there were higher levels of each following recovery from infection, compared with healthy children (Figure 1, A–C). Overall, IgG levels against all 3 *S*. *aureus* toxins were higher during convalescence, compared with during acute infection (Figure 1, D–F). This suggests that *S*. *aureus* infection elicited an antibody response in children. However, many children did not have an increase (<1.5-fold) in IgG levels from acute infection to convalescence (Hla: 41%, LukE: 38%, LukS-PV: 44%; Figure 1, D–F, red lines). Therefore, the antibody response to infection was highly variable in *S*. *aureus*–infected children.

We tested if antibody levels were associated with age or the severity of infection. As we previously reported, IgG levels during acute infection were associated with increasing age (Hla, *P* < 0.001; LukE, *P* = 0.003; LukS-PV, *P* = 0.004; Supplemental Figure 1, A–C; supplemental material available online with this article; https://doi.org/10.1172/jci.insight.173526DS1). There was a trend toward higher levels in children with invasive infection, but the differences were not significant (*P* = 0.2–0.4, Supplemental Figure 1, D–F). We next tested if the change in IgG levels from acute infection to convalescence differed based on age or the severity of infection. IgG levels against Hla, LukE, and LukS-PV increased following infection in children < 5 years or > 10 years, but not those between 5 and 10 years (Supplemental Figure 1, A–C). However, there were relatively few children in the 5- to 10-year-old age group, potentially masking a difference. Children with invasive and noninvasive infection had increases in IgG levels against Hla, LukE, and LukS-PV (Supplemental Figure 1, D–F). Hla-specific IgG1 levels largely reflected total IgG levels (Supplemental Figure 2), suggesting that the differences observed were not specific to any IgG subclass. However, we were able to quantify only Hla-specific IgG2 levels in the children with the highest Hla-specific total IgG levels and were unable to detect Hla-specific IgG3 or IgG4 levels (data not shown) because of limitations in serum volume. Together, these results suggest that neither age nor the severity of infection is associated with the ability of *S*. *aureus* infection to elicit an antibody response.

### Hla-specific IgG is associated with protection of immune cells from toxin-mediated killing.

**Figure 2:**
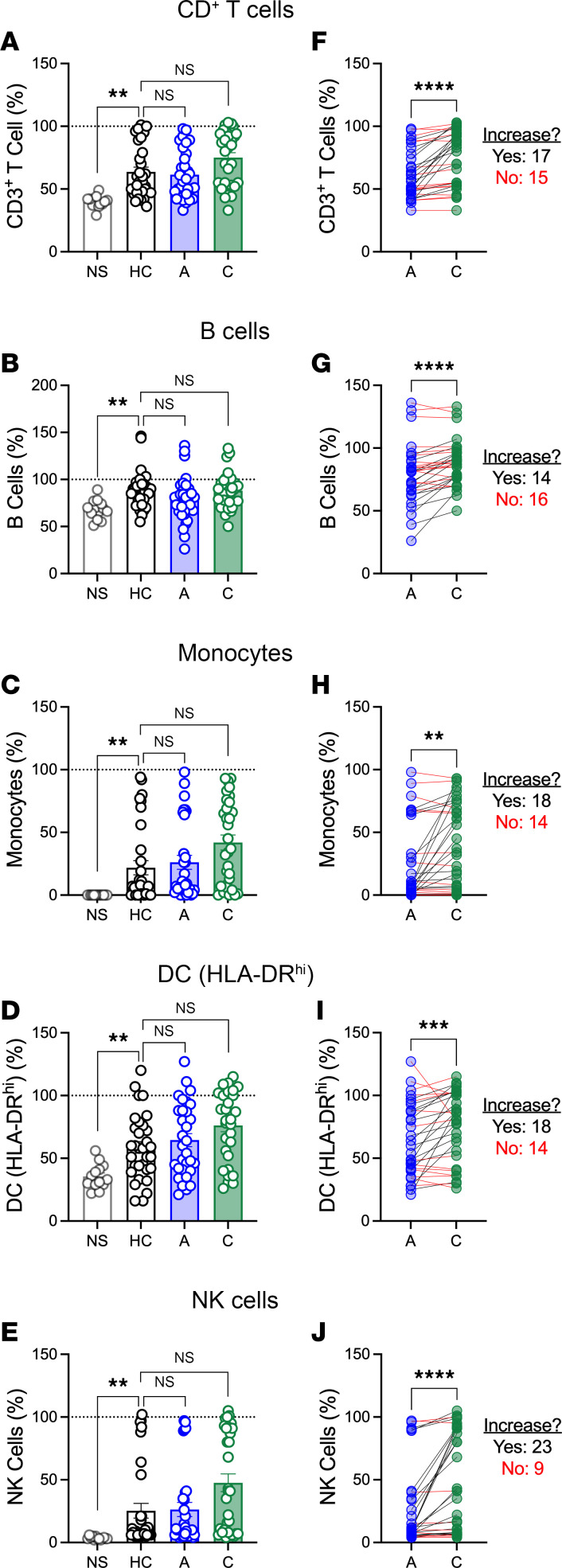
Protection of immune cells against toxin-mediated killing by acute and convalescent sera. PBMCs from healthy adults were incubated with *S*. *aureus* (USA300) supernatant and serum from study participants followed by quantification of live immune cells by flow cytometry (gating strategy, Supplemental Figure 3). (**A**–**E**) Protection of CD3^+^ T cells (**A**), B cells (**B**), monocytes (**C**), HLA-DR^hi^ dendritic cells (DC) (**D**), and NK cells (**E**) by serum from healthy children (HC), acutely infected children (“A”), and during convalescence (“C”). “NS” on *x* axes indicates no-serum controls. (**F**–**J**) Change in immune cell protection for individual study participants from acute infection to convalescence. Black lines indicate participants for whom immune cell survival increased ≥ 10%; red lines indicate participants for whom immune cell survival did not increase. Data are expressed as percentage immune cell survival, compared with no supernatant control (not shown) and presented as the median values with individual values superimposed on the plots. Data were compared by 1-way ANOVA with Kruskal-Wallis posttest (**A**–**E**) or Wilcoxon matched pairs signed rank test (**F**–**J**). **P < 0.01; ***P < 0.001; ****P < 0.0001.

We next determined if the increase in IgG levels during convalescence reflected improved antibody function by testing the ability of patient serum to inhibit *S*. *aureus* killing of immune cells. To accomplish this, we developed an ex vivo protection assay in which peripheral blood mononuclear cells (PBMCs) from healthy adults were coincubated with supernatant from *S*. *aureus* (USA300) and serum from healthy, acutely infected, or convalescing children. Following incubation, survival of immune cells (T cells, B cells, monocytes, dendritic cells [DCs], and natural killer [NK] cells) was quantified by flow cytometry (Supplemental Figure 3, gating strategy). In the absence of serum (no-serum controls; NS), *S*. *aureus* supernatant killed CD3^+^ T cells, monocytes, DCs (HLA-DR^hi^), and NK cells but less so B cells (Figure 2, A–E). Compared with no-serum controls, serum from healthy and infected children protected immune cells against *S*. *aureus*–mediated killing (Figure 2, A–E). Similar results were observed for CD4^+^ and CD8^+^ T cells and HLA-DR^mid^ DCs (data not shown). This killing was mediated largely by *sae*-regulated toxins (e.g., Hla, LukE, LukS-PV), because incubation with supernatant from an isogenic saeRS deletion mutant (*Δsae*), which does not express these toxins (21), failed to kill host immune cells, with the exception of monocytes, which had very low survival even after incubation with *Δsae* (Supplemental Figure 4). Validating the importance of toxins, there were no significant differences in immune cell killing by *Δsae* among healthy controls, acutely infected children, and convalescing children (Supplemental Figure 4). Importantly, the ability of serum to inhibit immune cell killing was stronger during convalescence, compared with during acute infection (Figure 2, F–J). This suggests that infection raised functional antibodies that inhibited toxin-mediated targeting of immune cells. However, as we observed with antibody levels, there was marked interindividual variability in the increase from acute infection to convalescence, and nearly half of children had less than 10% increase in protection of immune cells (T cells: 47%; B cells: 53%; monocytes: 44%; DCs: 44%; NK cells 28%; Figure 2, F–J, red lines).

**Figure 3:**
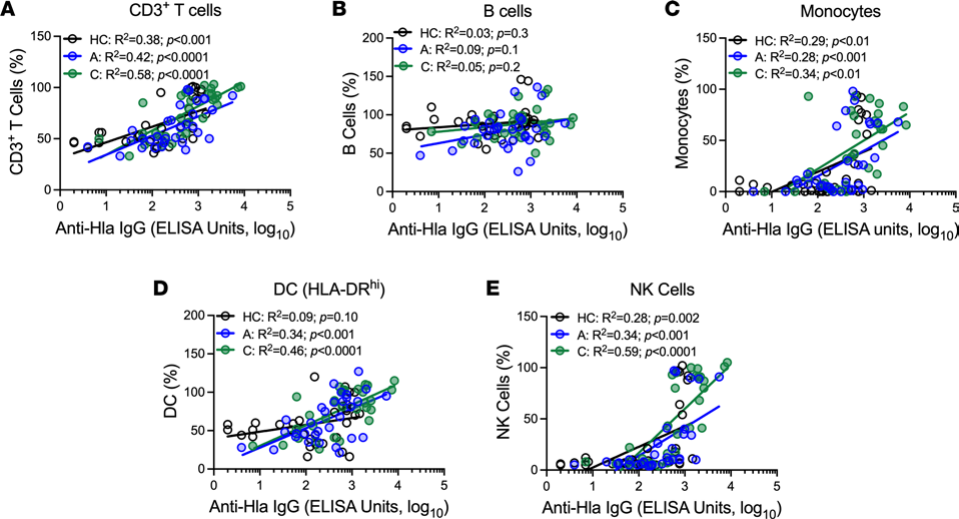
Hla-specific IgG levels correlate with protection of immune cells against toxin-mediated killing. Correlation of Hla-specific IgG levels (ELISA units, log_10_) with protection of T cells (**A**), B cells (**B**), monocytes (**C**), dendritic cells (**D**), and NK cells (**E**) for healthy children (HC), for acutely infected children (“A”), or during convalescence (“C”). Correlations were determined by linear correlation using log_10_-transformed IgG levels.

To identify which toxin-specific antibodies mediated protection of immune cells, we tested if Hla-, LukE-, or LukS-PV–specific IgG levels correlated with immune cell killing. In healthy children, there was a moderate correlation (*R*^2^ = 0.28–0.38) between anti-Hla IgG and protection of T cells (Figure 3A), monocytes (Figure 3C), and NK cells (Figure 3E) but no correlation for B cells (Figure 3B) or DCs (Figure 3D). In acutely infected children, there was a moderate correlation (*R*^2^ = 0.28–0.42) between Hla-specific IgG and survival of T cells (Figure 3A), monocytes (Figure 3C), DCs (Figure 3D), and NK cells (Figure 3E) but no correlation for B cells (Figure 3B). These results suggest that healthy and infected children had functional anti-toxin antibodies. Interestingly, there were stronger correlations (*R*^2^ = 0.46–0.59) during convalescence between anti-Hla IgG levels and protection of T cells (Figure 3A), DCs (Figure 3D), and NK cells (Figure 3E), compared with healthy or acutely infected children, but no differences for B cells (Figure 3B) or monocytes (Figure 3C). In contrast, there were weak correlations of LukE IgG levels with protection of T cells (*R*^2^ < 0.2), monocytes (*R*^2^ < 0.3), and NK cells (*R*^2^ < 0.2) in healthy and acutely infected children and a weak correlation of LukE IgG levels with protection of B cells (*R*^2^ < 0.3) and DCs (*R*^2^ < 0.15) only in infected children (Supplemental Figure 5A). Similarly, there were weak correlations (*R*^2^ < 0.3) of LukS-PV IgG levels with protection of T cells, B cells, monocytes, DCs, and NK cells only during convalescence (Supplemental Figure 5B). Together, these findings suggest that infection raises functional toxin-specific antibodies and point to a major role for Hla in protection of immune cells.

To test the specific role of Hla in immune cell killing, we modified our killing assay by culture of recombinant active Hla with participant serum (*N* = 10/group) before incubation with healthy adult PBMCs. As we observed with *S*. *aureus* supernatant–induced killing, convalescent serum best protected T cells, B cells, and NK cells against Hla-mediated toxicity (Supplemental Figure 6, A, B, and E), though there were no significant differences for monocytes and DCs (Supplemental Figure 6, C and D), likely due to the smaller sample size. There was also an increase in the ability to protect against Hla-mediated immune cell killing following recovery from infection in most, but not all, infected children (Supplemental Figure 6, F–J). There were strong correlations (*R*^2^ = 0.5–0.6) between anti-Hla IgG levels and protection of immune cells during convalescence but no or much weaker correlations during acute infection or in healthy children (Supplemental Figure 7). Taken together, these findings suggest that infection elicits strong, functional Hla-specific antibodies that protect immune cells against Hla-mediated killing.

### Impaired T cell responses during infection recover in a subset of infected children.

**Figure 4:**
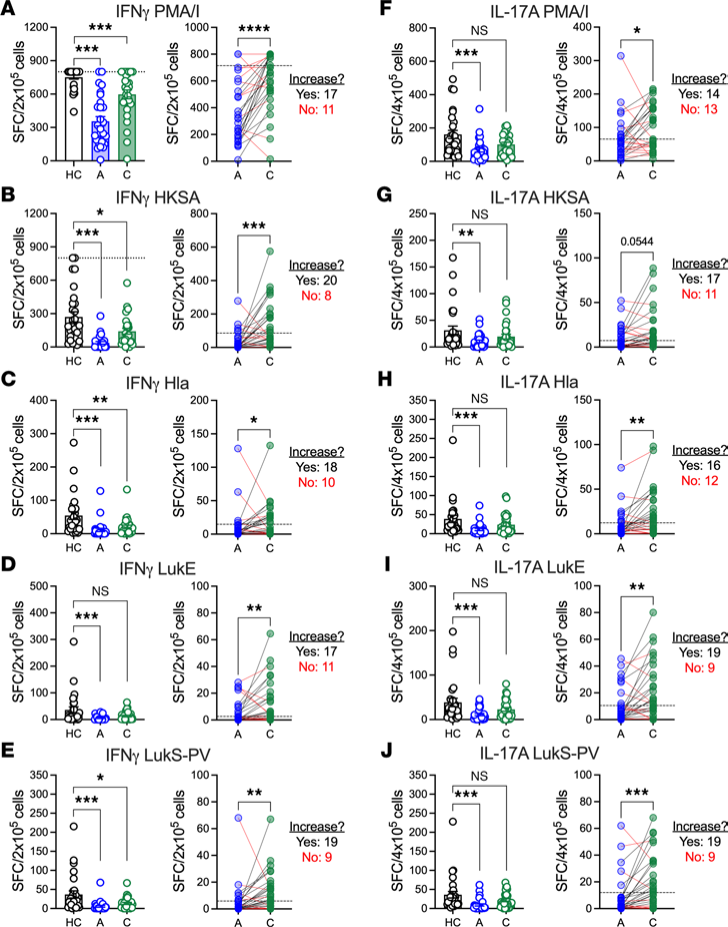
Effector T cell responses during acute infection and convalescence. Effector T cell responses were quantified in participant PBMCs (HC, healthy control; A, acute infection; C, convalescence) by IFN-γ or IL-17A ELISPOT following incubation with PMA/ionomycin (PMA/I), heat-killed *S*. *aureus* (HKSA), or purified Hla, LukE, or LukS-PV. (**A**–**E**, left panels) Quantification of IFN-γ–secreting cells in healthy children, during acute infection, or during convalescence following culture with PMA/I (**A**), HKSA (**B**), Hla (**C**), LukE (**D**), and LukS-PV (**E**). (**A**–**E**, right panels) Change in IFN-γ–secreting cells for individual study participants from acute infection to convalescence. (**F**–**J**, left panels) Quantification of IL-17A–secreting cells in healthy children, during acute infection, or during convalescence following culture with PMA/I (**F**), HKSA (**G**), Hla (**H**), LukE (**I**), and LukS-PV (**J**). (**F**–**J**, right panels) Change in IL-17A–secreting cell numbers for individual study participants from acute infection to convalescence. (**A**–**J**, right panels) Black lines indicate participants for whom cytokine-secreting cells increased ≥ 1.5-fold; red lines indicates participants for whom cytokine-secreting cells did not increase (< 1.5-fold). Dashed lines indicate the 25th percentile of cytokine-secreting cells for healthy controls. Data are presented as the median values with individual values superimposed on the plots and were compared by 1-way ANOVA with Kruskal-Wallis posttest (**A**–**J**, left panels) or Wilcoxon matched pairs signed rank test (**A**–**J**, right panels). *P < 0.05; **P < 0.01; ***P < 0.001; ****P < 0.0001. SFC, spot-forming colonies.

We next quantified functional T cell IL-17A and IFN-γ responses during acute *S*. *aureus* infection and convalescence in 33 healthy and 28 infected children (5 participants did not have PBMC samples) by PBMC ELISPOT following stimulation with PMA/ionomycin (nonspecific T cell activation), heat-killed *S*. *aureus* (HKSA), or purified Hla_H35L_, LukE, or LukS-PV. As we previously reported, there were decreased numbers of IFN-γ–secreting (Figure 4, A–E, left) and IL-17A–secreting (Figure 4, F–J, left) cells during acute *S*. *aureus* infection following culture with PMA/ionomycin, HKSA, Hla, LukE, and LukS-PV, compared with healthy children (HC vs. A). For IFN-γ, there remained fewer cytokine-secreting cells during convalescence, compared with healthy children, following incubation with PMA/ionomycin, HKSA, Hla, and LukS-PV but not LukE (Figure 4, A–E, left; HC vs. C). In contrast, there were no significant differences between convalescing and healthy children for IL-17A (Figure 4, E–J, left; HC vs. C). These findings suggest that IL-17A responses recovered more fully than did IFN-γ responses.

We next directly compared acute and convalescent T cell responses. There were more IFN-γ–secreting cells during convalescence, compared with during acute infection, for all stimuli (Figure 4, A–E, right; A vs. C). Similarly, there were more IL-17A–secreting cells during convalescence, compared with during acute infection, following culture with PMA/ionomycin, Hla, LukE, and LukS-PV, and a trend toward stronger convalescent responses following culture with HKSA. However, as we observed with antibody levels and function, nearly one-third to one-half of children did not have recovery (increase of ≥1.5-fold) of IFN-γ (PMA/I: 40%; HKSA: 29%; Hla: 36%; LukE: 40%; LukS-PV: 32%; Figure 4, A–E, right, red lines) or IL-17A (PMA/I: 46%; HKSA: 40%; Hla: 43%; LukE: 32%; LukS-PV: 32%; Figure 4, F–J, right, red lines) responses. While some children whose T cell responses failed to increase already had high numbers of cytokine-secreting cells during acute infection, many children did not recover their T cell responses above the 25th percentile (dashed lines in Figure 4, right panels) of those of healthy children (IFN-γ: 36%–64%; IL-17A: 36%–46%). Taken together, these findings demonstrate that many children do not recover *S*. *aureus*–specific T cell responses, and among those who do, many continue to have fewer cytokine-secreting T cells during convalescence, compared with healthy children.

### Convalescent toxin-specific IgG levels correlate with enhanced protection of immune cells.

**Figure 5:**
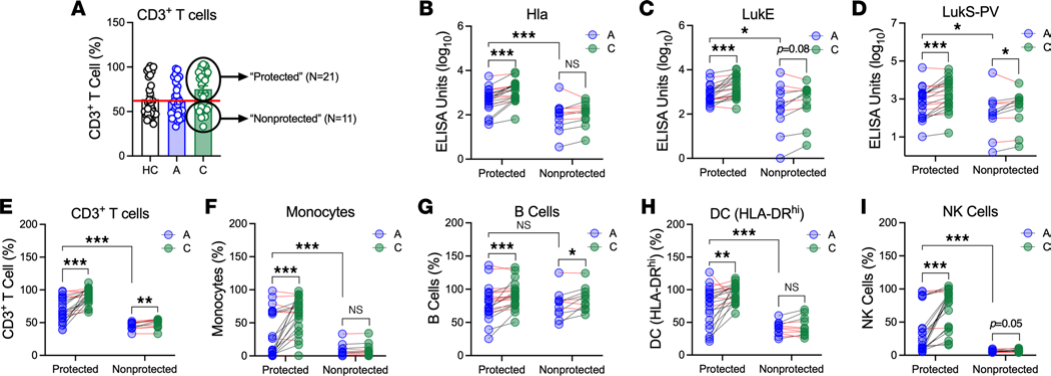
Children whose serum protects T cells against toxin-mediated killing have stronger increases in IgG levels and immune cell protection. (**A**) Based on the data in Figure 2A, infected children were separated into those whose convalescent serum strongly protected T cells (≥ 60% survival, “protected,” *N* = 21) and those whose convalescent serum less strongly protected T cells (< 60% survival, “nonprotected,” *N* = 11). (**B**–**D**) Levels of Hla-, LukE-, and LukS-PV–specific IgG during acute infection and convalescence in children whose serum more strongly protected T cells and those whose serum less strongly protected. (**E**–**I**) Protection of CD3^+^ T cells, monocytes, B cells, HLA-DR^hi^ DCs, and NK cells during acute infection and convalescence in children whose serum more strongly protected T cells and those whose serum less strongly protected. Data are expressed as arbitrary ELISA units (log_10_) (**B**–**D**) or percentage immune cell survival, compared with no supernatant control (not shown) (**A** and **E**–**I**). Data were compared using Wilcoxon matched pairs signed rank test (A vs. C) or Mann-Whitney *U* test (P vs. NP). *P < 0.05; **P < 0.01; ***P < 0.001.

The similarly high percentages of children who failed to increase antibody levels (38%–44%) or function (28%–50%) and those who did not recover their effector T cell responses (29%–46%) suggested that elicited antibody and recovery of T cell impairment are linked. We hypothesized that stronger anti-toxin antibody responses to infection associated with better protection of antigen-presenting cells (APCs) and T cells. To test this hypothesis, we leveraged our observation that there were 2 distinct populations of children in the context of the ability of convalescent serum to protect T cells from toxin-mediated killing (Figure 2A). We observed that convalescent serum from 21 infected children strongly protected CD3^+^ T cells (≥ 60% survival, median value for healthy controls, Figure 5A), whereas serum from 11 infected children did not (< 60%, Figure 5A). Similar results were observed for CD4^+^ and CD8^+^ T cell subsets (data not shown). We therefore analyzed these 2 groups of children, termed “protected” (those whose serum more strongly protected T cells) and “nonprotected” (Figure 5A).

Children whose serum better protected T cells from toxin-mediated killing were older and more likely to be White, compared with those whose serum was less protective (Supplemental Table 1). However, there were no differences between the groups in terms of the severity of infection or whether the infecting isolate was MRSA or MSSA. We first compared anti-toxin IgG levels between children with protective and nonprotective serum. Not surprisingly, because Hla-specific IgG levels correlated with stronger protection of T cells against bacterial killing, we found that Hla-specific IgG levels were much higher in protected versus nonprotected sera during both acute infection and convalescence (Figure 5B). LukE and LukS-PV–specific IgG levels were also higher in protected versus nonprotected sera (Figure 5, C and D). Interestingly, children whose serum better protected T cells had an increase in anti-toxin IgG levels from acute infection to convalescence, whereas those whose serum less potently protected T cells did not, with the exception of a small increase in LukS-PV–specific IgG (Figure 5, B–D). Consistent with higher IgG levels, protective acute and convalescent serum more strongly protected T cells, monocytes, DCs, and NK cells, but not B cells, from toxin-mediated killing (Figure 5, E–I). Importantly, among children whose serum more strongly protected immune cells from toxin-mediated killing, there was an increase in protection from acute infection to convalescence, consistent with infection raising a functional antibody response in these children (Figure 5, E–I). In contrast, children whose serum less strongly protected immune cells failed to increase protection of immune cells from acute infection to convalescence, suggesting a failure of infection to elicit a functional antibody response (Figure 5, E–I). Together, these results suggest that the ability of convalescent serum to protect immune cells against toxin-mediated killing may identify children with a stronger functional antibody response to *S*. *aureus* infection.

### Children with serum that more strongly protects immune cells against toxin-mediated killing have greater recovery of T cell impairment.

**Figure 6:**
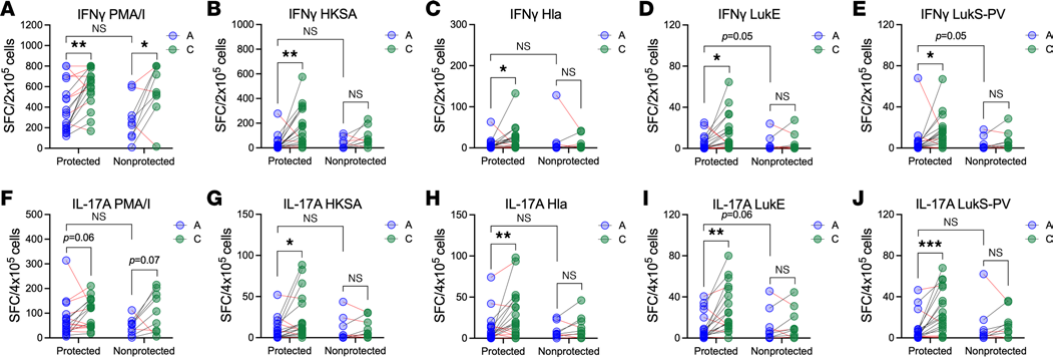
Children whose serum protects T cells against toxin-mediated killing have stronger recovery of impaired *S*. *aureus*–specific T cell responses. As described in Figure 5, infected children were separated into those whose convalescent serum strongly protected T cells (“protected,” *N* = 21) and those whose convalescent serum less strongly protected T cells (“nonprotected,” *N* = 11). (**A**–**E**) Number of IFN-γ–secreting cells during acute infection and convalescence following culture with PMA/I, HKSA, Hla, LukE, or LukS-PV in children whose serum more strongly protected T cells and those whose serum less strongly protected. (**F**–**J**) Number of IL-17A–secreting cells during acute infection and convalescence following culture with PMA/I, HKSA, Hla, LukE, or LukS-PV in children whose serum more strongly protected T cells and those whose serum less strongly protected. Data were compared using Wilcoxon matched pairs signed rank test (A vs. C) or Mann-Whitney *U* test (P vs. NP). *P < 0.05; **P< 0.01; ***P < 0.001.

We next hypothesized that anti-toxin antibody protection of APCs and T cells against toxin-mediated killing permits recovery of T cell impairment following infection. To test this hypothesis, we compared IFN-γ and IL-17A responses by ELISPOT between children with protective and nonprotective serum. Surprisingly, there were no significant differences in the severity of impairment of IFN-γ–secreting (Figure 6, A–E) or IL-17A–secreting (Figure 6, F and G) cells during acute infection between children with protective and nonprotective serum, though there were trends toward stronger suppression of LukE-specific (IFN-γ and IL-17A, Figure 6, D and I) and LukS-PV–specific (IFN-γ, Figure 6E) responses in those with nonprotective serum. Following stimulation with PMA/ionomycin, convalescent children had more IFN-γ–secreting cells (Figure 6A) and a trend toward more IL-17A–secreting cells (Figure 6F), compared with during acute infection, regardless of how strongly their serum protected immune cells. This suggests that nonspecific T cell responses recovered in both groups. In contrast, there were significantly higher numbers of IFN-γ–secreting cells following stimulation with HKSA, Hla, LukE, or LukS-PV during convalescence only in children with protective serum (Figure 6, B–E). Similarly, there was recovery of *S*. *aureus*– and antigen-specific IL-17A responses in children with protective serum but not those with nonprotective serum (Figure 6, G–J). Together, these results suggest that antibody and T cell responses to infection are linked. Specifically, these data suggest that higher anti-toxin antibody levels “protect” APCs and T cells and contribute to enhanced recovery of T cell responses following infection. Interestingly, this phenomenon was observed only for *S*. *aureus*– and antigen-specific T cell responses; recovery of nonspecific T cell responses occurred equally well in both groups.

## Discussion

In this study, we found that *S*. *aureus* infection during childhood elicits pathogen-specific functional antibody and T cell responses. Toxin-specific antibody levels increased following *S*. *aureus* infection, but the individual responses were highly variable; nearly 40% of children did not have an increase in antibody levels. Hla-specific (but less so LukE or LukS-PV) antibody levels correlated with protection of immune cells from toxin-mediated killing, and this correlation was strongest during convalescence, consistent with a functional antibody response. Similarly, there was an increase in *S*. *aureus*–specific cytokine-secreting T cells following infection, indicative of expansion or recovery of impaired T cell responses during acute infection. However, as with antibody levels and function, improvement in T cell function was observed in only 60%–70% of children. Children whose convalescent serum more strongly protected T cells from toxin-mediated killing had a greater increase in antibody levels and stronger improvement of T cell responses during convalescence. Taken together, these findings demonstrate that *S*. *aureus* infection is immunogenic and suggest that immunity raised by infection may be protective. However, there is marked heterogeneity among individuals. These findings also suggest that toxin-specific antibodies “protect” immune cells, thereby promoting expansion or recovery of *S*. *aureus*–specific T cell responses.

Our findings that antibody levels increase from acute infection to convalescence are consistent with those of Thomsen et al., who found that IgG levels against a number of exotoxins increased during childhood *S*. *aureus* infection (22). Interestingly, however, they did not find an increase in LukE-specific IgG. Their study enrolled mainly children with invasive infection (90%), so this may contribute to the discrepancy. In contrast with our findings that IgG levels increased in children with invasive or noninvasive infection, Fritz et al. reported that Hla-specific IgG levels increased following infection only in children with invasive infection; children with primary or recurrent SSTI had a decrease in IgG levels (6). The reasons for these discrepancies are not clear, but differences in patient populations, timing of sampling, and methods for antibody quantification may contribute. While the overall antibody levels increased from acute infection to convalescence in our cohort, we found marked individual heterogeneity in the response to infection; nearly 40% of children did not have an increase in antibody levels. Surprisingly, these differences do not appear to be attributable to age or the severity of infection. While we found that IgG1 levels largely reflected total IgG levels, we did not have sufficient serum volume to compare other IgG subclasses, in the context of antibody levels or their specific roles in toxin neutralization. Taken together, the current and reported studies suggest that individual variability in immune responses to infection merits further study.

Thomsen et al. found that LukAB-mediated toxicity toward neutrophils was inhibited by serum from children with *S*. *aureus* infection, suggesting that antibodies raised by infection are functional (22). Our findings extend this idea by demonstrating that convalescent sera more strongly protect immune cells important in the adaptive immune response, namely T cells, B cells, monocytes, DCs, and NK cells, compared with acute sera. Moreover, we found strong correlations with Hla IgG levels and protection of T cells, monocytes, DCs, and NK cells from toxin-mediated killing. This is consistent with widespread expression of the Hla receptor ADAM10 on host immune cells and explains the sensitivity of these cells to Hla (reviewed in ref. 23). We were surprised to find weak or no correlations between LukE-specific IgG levels and protection of T cells, DCs, or monocytes, because LukE targets these cells by binding CCR5 (24). In contrast, LukS-PV binds human monocytes but not T cells via C5a receptors (25). Together, these findings suggest that Hla-specific IgG plays a dominant role in protection of cells necessary for an adaptive immune response. This hypothesis is supported by our observations that protection of immune cells by patient serum correlates with stronger recovery of functional effector T cell responses. Indeed, we verified that Hla specifically targets these immune cells, and Hla-specific IgG levels correlated with protection against Hla-mediated toxicity, most prominently during convalescence. Thus, these findings may explain the observations of Fritz et al. that Hla-specific antibody is associated with protection against recurrent infection (6). It is possible, however, that antibodies against the “F” components of these toxins or other bicomponent leukotoxins (e.g., LukAB, HlgAB, HlgCB) (26, 27) not tested in this study may be co-elicited with Hla IgG and contribute to immune cell protection. Future studies will test this possibility.

We previously reported that nonspecific and *S*. *aureus*–specific IL-17A and IFN-γ responses were strongly impaired during *S*. *aureus* infection (20). These findings extend our prior work by demonstrating that effector T cell responses are stronger following infection, at least in a subset of children. As with antibody levels, this was not attributable to the severity of infection or age. There are at least 2 possibilities to explain these findings. First, weaker *S*. *aureus*–specific T cell responses may predispose children to infection. If this is the case, the increase in T cell responses following infection may represent expansion of *S*. *aureus*–specific T cells elicited by infection. However, infected children have similar antibody levels, compared with healthy children, arguing against a cryptic *S*. *aureus*–specific “hole” in the immune response. Conversely, we propose that T cell responses are acutely suppressed during infection and recover thereafter. As we observed with antibody levels, the response to infection was highly variable, as only 60%–70% of children increased their T cell responses. Moreover, some children, despite increasing their T cell responses compared with during acute infection, had persistently low convalescent T cell responses. We speculate that convalescent *S*. *aureus*–specific T cell responses can predict susceptibility to recurrent infection; longitudinal studies to address this possibility are ongoing.

Our findings suggest a link between toxin-specific antibody levels and expansion/recovery of T cells. Specifically, these findings prompt the hypothesis that toxin-specific antibodies (most likely Hla-specific) protect APCs and T cells, thereby permitting the expansion or recovery of T cell responses following infection. Indeed, children whose convalescent serum protected immune cells less effectively against toxin-mediated killing also were more likely to (i) start with lower antibody levels during acute infection and (ii) fail to increase antibody levels in response to infection. These lower antibody levels correlated with (i) inferior protection of immune cells during acute infection and (ii) a failure to increase protection of immune cells during convalescence, indicating a failure to elicit functional anti-toxin antibody. Importantly, these same children, despite no differences in T cell suppression during acute infection, failed to recover or expand their T cell responses during convalescence. Together, these findings suggest that (i) T cell suppression is a feature of acute *S*. *aureus* infection regardless of preexisting antibody levels or severity of infection and (ii) recovery or expansion of impaired T cell responses following infection is promoted by antibodies that prevent toxin-mediated killing of APCs and T cells. Interestingly, children recovered their global T cell responses independent of whether their serum protected immune cells, suggesting that toxins specifically impair *S*. *aureus*–specific T cell responses.

We acknowledge several limitations of this study. First, this is a small cohort at a single center, and the majority of study participants were White and male; additional study is necessary to enable generalization of the results. Moreover, without large population-based studies, it is impossible to know the immunological “baseline” of the infected children. Similarly, the study was not powered for subgroup analysis; thus, any such analyses were treated as pilot studies for additional investigation. Second, the ELISPOT assay is not able to discriminate between impairment at the level of the APC or T cell; ongoing studies are focused on identifying the specific level of impairment. Given our findings that toxins killed both APCs and T cells, we speculate that APC and T cell function is each impaired during infection and together contribute to suppressed functional T cell responses. Similarly, the ELISPOT assay cannot identify the phenotype of the cytokine-secreting cells. This is particularly important in the context of *S*. *aureus*–specific memory T cells. This study was not designed to determine if antibody levels impact the outcome of acute infection or protection against recurrent infection; these are 2 important future directions to demonstrate the clinical relevance of this work. This is of particular relevance because Adhikari et al. reported that, among adults with *S*. *aureus* bacteremia, higher anti-toxin antibody levels were associated with a lower incidence of sepsis (28). While Hla-targeted strategies to prevent and treat *S*. *aureus* infections remain promising, a phase II trial of a monoclonal antibody against Hla failed to prevent ventilator-associated pneumonia among mechanically ventilated patients (29). Finally, longitudinal studies are necessary to determine if impairment of acute or convalescent immune responses can predict the risk of recurrent infection.

These findings have several potential implications. First, *S*. *aureus* infection elicits highly variable antibody and T cell responses in children. While age may play a role in this variability, it is likely that host genetics are also important. Therefore, future studies should identify genetic variants that determine the response to infection. Second, these findings suggest that toxin-specific antibodies may determine the impact of infection on elicited T cell responses. Given the high frequency of *S*. *aureus*–specific memory T cells in adults (30), a composite of antigen-specific antibodies and protective T cell responses may both serve as a correlate of protection and inform ideal target mechanisms of protection for future vaccines. This is consistent with mouse models of *S*. *aureus* infection, in which both antibodies and T cells contribute to protection (31–35).

In summary, we observed that *S*. *aureus* infection elicits an immune response in children, but the response to infection is highly variable. These findings also highlight a potential role for Hla-specific antibodies in protection of immune cells against toxin-mediated killing and may provide a link between protective antibodies and recovery of impaired T cell responses that ultimately determine protective immunity against recurrent infection.

## Methods

### Study design.

This is a single-center prospective observational study. Healthy children were enrolled in outpatient settings. Hospitalized children (6 months to 21 years old) were identified by positive *S*. *aureus* culture or by clinical presentation with subsequent culture confirmation. Exclusion criteria included documented immunodeficiency, receipt of immunosuppressive medications within 2 months (excluding ≤ 5 days of corticosteroids), or receipt of an Ig-based product within 6 months. Clinical information was entered into a REDCap database. Blood samples were collected within 3 days of positive culture. Convalescent samples were collected 4–6 weeks after enrollment. Blood was collected in mononuclear cell preparation (CPT) and serum separation (SST) tubes (Becton Dickinson). PBMCs were frozen in liquid nitrogen and serum was frozen at –80°C.

### Antibody quantification.

Antibodies against Hla, LukE, and LukS-PV were quantified by ELISA as previously reported (20). Briefly, 96-well plates (Costar, Corning) were coated with 4 μg/mL of purified antigen. Serial dilutions of a standardized serum stock prepared from pooled healthy adult serum were included on each plate to minimize variability. Serial dilutions of participant sera were added (all samples plated in duplicate), followed by quantification of IgG using alkaline phosphatase–conjugated goat anti-human IgG (Kirkegaard and Perry Labs) or horseradish peroxidase–conjugated anti-human IgG1 (Invitrogen) and AP substrate *p*-nitrophenyl phosphate (MilliporeSigma) or tetramethylbenzidine dihydrochloride (Invitrogen). OD_405_ was measured using a GENios spectrophotometer (Tecan). IgG levels were quantified as arbitrary ELISA units. Data were considered satisfactory if: (a) the *R*^2^ of the standard curve of each plate was ≥ 0.995, (b) the coefficient of variance between duplicates was < 20%, (c) the OD_405_ was between 0.5 and 2.5 and within the standard range, and (d) blank wells had an OD_405_ ≤ 0.10. If these criteria were not met, then the sample was repeated.

### Quantification of immune cell killing.

PBMCs were thawed, and an aliquot was separated for cell counting. *S*. *aureus* supernatant (wild-type USA300 “923” and isogenic *Δsae*) (21) was prepared from planktonic cultures (OD_600_ ~1.4). PBMCs (1 × 10^6^) were incubated with *S*. *aureus* supernatant (1:6 dilution) and participant serum (1:10 dilution, corrected for total IgG 200 mg/dL) for 3 hours at 37°C. Our flow cytometry strategy has been reported (20). For direct Hla toxicity studies, recombinant active Hla (0.2 μg, Abcam) (36) was incubated with participant serum for 30 minutes, followed by 3-hour incubation with PBMCs. Following incubation, cells were washed with phosphate-buffered saline, stained with Live/Dead stain (Becton Dickinson), washed with fluorescence-activated cell sorting (FACS) buffer (Becton Dickinson), and blocked with Human FcR Block (Becton Dickinson). Surface staining was performed with anti-CD3 (UCHT1), anti-CD4 (L200), anti-CD8 (SK1), anti-CD19 (HIB19), anti-CD45 (HI30), anti-CD16 (3G8), anti–HLA-DR (L243), anti-CD56 (NCAM1), anti-CD14 (M5E2), and anti-CD11c (Bu15). Staining was performed in Brilliant stain buffer (Becton Dickinson) at 4°C for 30 minutes. Antibodies were purchased from Becton Dickinson (anti-CD56 and anti-CD4) and BioLegend (all others). Cells were washed with FACS buffer and fixed overnight at 4°C in 4% paraformaldehyde. Cells were washed and reconstituted in FACS buffer with the addition of counting beads. Flow cytometry was performed using an LSRFortessa flow cytometer (Becton Dickinson). Data were analyzed using Flowjo.

### Quantification of effector T cell responses.

Our approach to quantification of effector T cell responses has been reported (20). Briefly, human IFN-γ and IL-17A T cell ELISPOT kits (U-Cytech) were used, and 96-well plates were coated with anti–IFN-γ or anti–IL-17A antibody, followed by the addition of thawed PBMCs (2 × 10^5^/well for IFN-γ; 4 × 10^5^/well for IL-17A). PMA and ionomycin, HKSA (USA300, 5 × 10^5^ CFU/well), or purified Hla_H35L_, LukE, or LukS-PV (20 μg/mL) were added. Samples were incubated at 37°C for 40 hours, followed by washing and addition of biotin-labeled detection antibodies (part of the kits). Each sample was plated in duplicate, and negative controls were incubated with media alone (no antigen). Samples were repeated if the negative control had ≥ 5 spots/well or if the coefficient of variance was ≥ 20% between replicates. Spots were developed using avidin horseradish peroxidase (eBioscience) and 3-Amino-9-ethylcarbazole substrate solution (Becton Dickinson) and counted using an Immunospot series 1 analyzer (Cellular Technology).

### Sex as a biological variable.

Male and female children were enrolled in this study. Infected children were enrolled in a convenience sample; thus, there were no prespecified design constraints for the numbers of male or females. Healthy children were matched with infected children by biological sex.

### Statistics.

Differences between participant groups were evaluated by Fisher exact and Wilcoxon rank sum tests. Antibody levels were log_10_-transformed for all analyses. Differences in antibody levels, serum protection against immune cell killing, and T cell responses between healthy and infected children were evaluated using the Wilcoxon rank sum exact test, 1-way ANOVA with Kruskal-Wallis posttest, or Mann-Whitney *U* test. Differences between acutely infected and convalescing children were evaluated using the Wilcoxon matched pairs signed rank test. Multivariable linear regression was used to evaluate differences between groups while accounting for age. The relationship between age and antibody levels was evaluated graphically with a loess curve and statistically with polynomial regression. Differences were considered significant if *P* < 0.05. All analyses were conducted using GraphPad Prism or R for Statistical Computing.

### Study approval.

This study was approved by the Institutional Review Board at Nationwide Children’s Hospital (IRB17-01176). Written informed consent (and assent when applicable) was obtained prior to enrollment.

### Data availability.

Supporting Data Values associated with the main manuscript and supplement, including values for all data points, are provided in the Supplemental Data Values file. Additional deidentified data may be available by written request.

## Author contributions

MK, ZL, UC, WP, and CPM designed the study. MK, ZL, UC, and WP conducted experiments and acquired data. MK, ZL, UC, WP, and RA analyzed data. AW and JP screened and enrolled study participants and acquired samples. MK and CPM wrote the manuscript. All authors read and revised the manuscript.

## Supplementary Material

Supplemental data

ICMJE disclosure forms

Supporting data values

## Figures and Tables

**Table 1:**
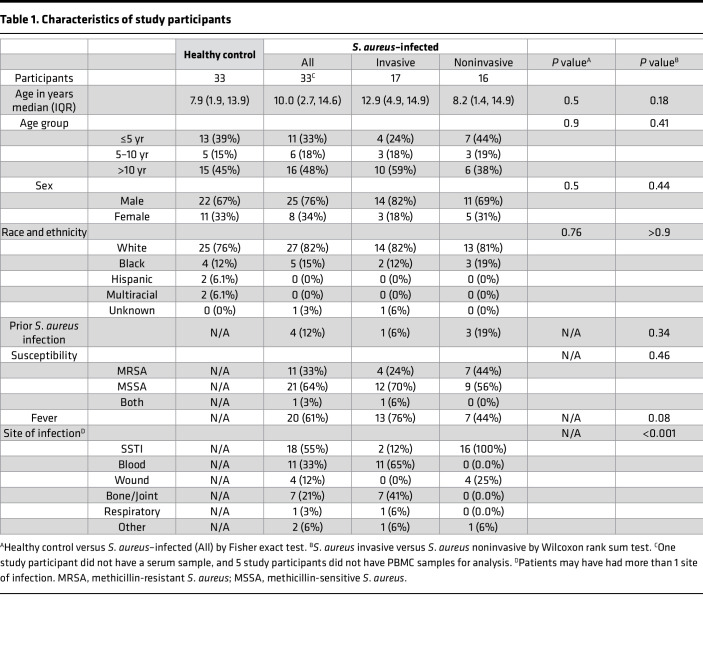
Characteristics of study participants

## Version 1 01/18/2024

In-Press Preview

## Version 2 02/22/2024

Electronic publication

## References

[1] David MZ, Daum RS (2010). Community-associated methicillin-resistant *Staphylococcus aureus*: epidemiology and clinical consequences of an emerging epidemic. Clin Microbiol Rev.

[2] Kourtis AP (2019). *Vital Signs*: epidemiology and recent trends in methicillin-resistant and in methicillin-susceptible *Staphylococcus aureus* bloodstream infections - United States. MMWR Morb Mortal Wkly Rep.

[3] Suaya JA (2014). Incidence and cost of hospitalizations associated with Staphylococcus aureus skin and soft tissue infections in the United States from 2001 through 2009. BMC Infect Dis.

[4] Alegre ML (2016). Impact of Staphylococcus aureus USA300 colonization and skin infections on systemic immune responses in humans. J Immunol.

[5] Kluytmans J (1997). Nasal carriage of Staphylococcus aureus: epidemiology, underlying mechanisms, and associated risks. Clin Microbiol Rev.

[6] Fritz SA (2013). A serologic correlate of protective immunity against community-onset Staphylococcus aureus infection. Clin Infect Dis.

[7] Miller LG (2015). Staphylococcus aureus skin infection recurrences among household members: an examination of host, behavioral, and pathogen-level predictors. Clin Infect Dis.

[8] Jahantigh HR (2022). The candidate antigens to achieving an effective vaccine against Staphylococcus aureus. Vaccines (Basel).

[9] Fowler VG (2013). Effect of an investigational vaccine for preventing Staphylococcus aureus infections after cardiothoracic surgery: a randomized trial. JAMA.

[10] Hassanzadeh H (2023). Efficacy of a 4-antigen *Staphylococcus aureus* vaccine in spinal surgery: the *Staphylococcus aureus* surgical inpatient vaccine efficacy (STRIVE) randomized clinical trial. Clin Infect Dis.

[11] Fattom A (2015). Efficacy profile of a bivalent Staphylococcus aureus glycoconjugated vaccine in adults on hemodialysis: phase III randomized study. Hum Vaccin Immunother.

[12] Salgado-Pabon W, Schlievert PM (2014). Models matter: the search for an effective Staphylococcus aureus vaccine. Nat Rev Microbiol.

[13] Tsai CM (2022). Non-protective immune imprint underlies failure of *Staphylococcus aureus* IsdB vaccine. Cell Host Microbe.

[14] Teymournejad O (2023). Toxin expression during *Staphylococcus aureus* infection imprints host immunity to inhibit vaccine efficacy. NPJ Vaccines.

[15] Hermos CR (2010). High levels of antibody to panton-valentine leukocidin are not associated with resistance to Staphylococcus aureus-associated skin and soft-tissue infection. Clin Infect Dis.

[16] Radke EE (2018). Hierarchy of human IgG recognition within the Staphylococcus aureus immunome. Sci Rep.

[17] Dryla A (2005). Comparison of antibody repertoires against Staphylococcus aureus in healthy individuals and in acutely infected patients. Clin Diagn Lab Immunol.

[18] Verkaik NJ (2009). Anti-staphylococcal humoral immune response in persistent nasal carriers and noncarriers of Staphylococcus aureus. J Infect Dis.

[19] Rigat F (2019). Retrospective identification of a broad IgG repertoire differentiating patients with S. aureus skin and soft tissue infections from controls. Front Immunol.

[20] Li Z (2021). Impaired T lymphocyte responses during childhood Staphylococcus aureus infection. J Infect Dis.

[21] Montgomery CP (2010). Importance of the global regulators Agr and SaeRS in the pathogenesis of CA-MRSA USA300 infection. PLoS One.

[22] Thomsen IP (2014). Children with invasive *Staphylococcus aureus* disease exhibit a potently neutralizing antibody response to the cytotoxin LukAB. Infect Immun.

[23] Seilie ES, Bubeck Wardenburg J (2017). Staphylococcus aureus pore-forming toxins: the interface of pathogen and host complexity. Semin Cell Dev Biol.

[24] Alonzo F (2013). CCR5 is a receptor for Staphylococcus aureus leukotoxin ED. Nature.

[25] Spaan AN (2013). The staphylococcal toxin Panton-Valentine leukocidin targets human C5a receptors. Cell Host Microbe.

[26] Berends ETM (2019). Staphylococcus aureus impairs the function of and kills human dendritic cells via the LukAB toxin. mBio.

[27] Spaan AN (2014). The staphylococcal toxins γ-haemolysin AB and CB differentially target phagocytes by employing specific chemokine receptors. Nat Commun.

[28] Adhikari RP (2012). Lower antibody levels to Staphylococcus aureus exotoxins are associated with sepsis in hospitalized adults with invasive S. aureus infections. J Infect Dis.

[29] Francois B (2021). Efficacy and safety of suvratoxumab for prevention of Staphylococcus aureus ventilator-associated pneumonia (SAATELLITE): a multicentre, randomised, double-blind, placebo-controlled, parallel-group, phase 2 pilot trial. Lancet Infect Dis.

[30] Kolata JB (2015). The fall of a dogma? Unexpected high T-cell memory response to Staphylococcus aureus in humans. J Infect Dis.

[31] Cho JS (2010). IL-17 is essential for host defense against cutaneous Staphylococcus aureus infection in mice. J Clin Invest.

[32] Kennedy AD (2010). Targeting of alpha-hemolysin by active or passive immunization decreases severity of USA300 skin infection in a mouse model. J Infect Dis.

[33] Montgomery CP (2014). Protective immunity against recurrent Staphylococcus aureus skin infection requires antibody and interleukin-17A. Infect Immun.

[34] Dillen CA (2018). Clonally expanded γΔ T cells protect against Staphylococcus aureus skin reinfection. J Clin Invest.

[35] Brown AF (2015). Memory Th1 cells are protective in invasive Staphylococcus aureus infection. PLoS Pathog.

[36] Tsuiji M (2019). Selective cytotoxicity of staphylococcal α-hemolysin (α-toxin) against human leukocyte populations. Biol Pharm Bull.

