# Supplementary material for: Toxin-neutralizing Abs are associated with improved T cell function following recovery from Staphylococcus aureus infection
Source: JCI Insight. 2024 Jan 18;9(4):e173526. doi: 10.1172/jci.insight.173526 (PMC11143924; doi:10.1172/jci.insight.173526)
Supplement: Supplemental data [file jciinsight-9-173526-s091.pdf]

SUPPLEMENTARY MATERIAL

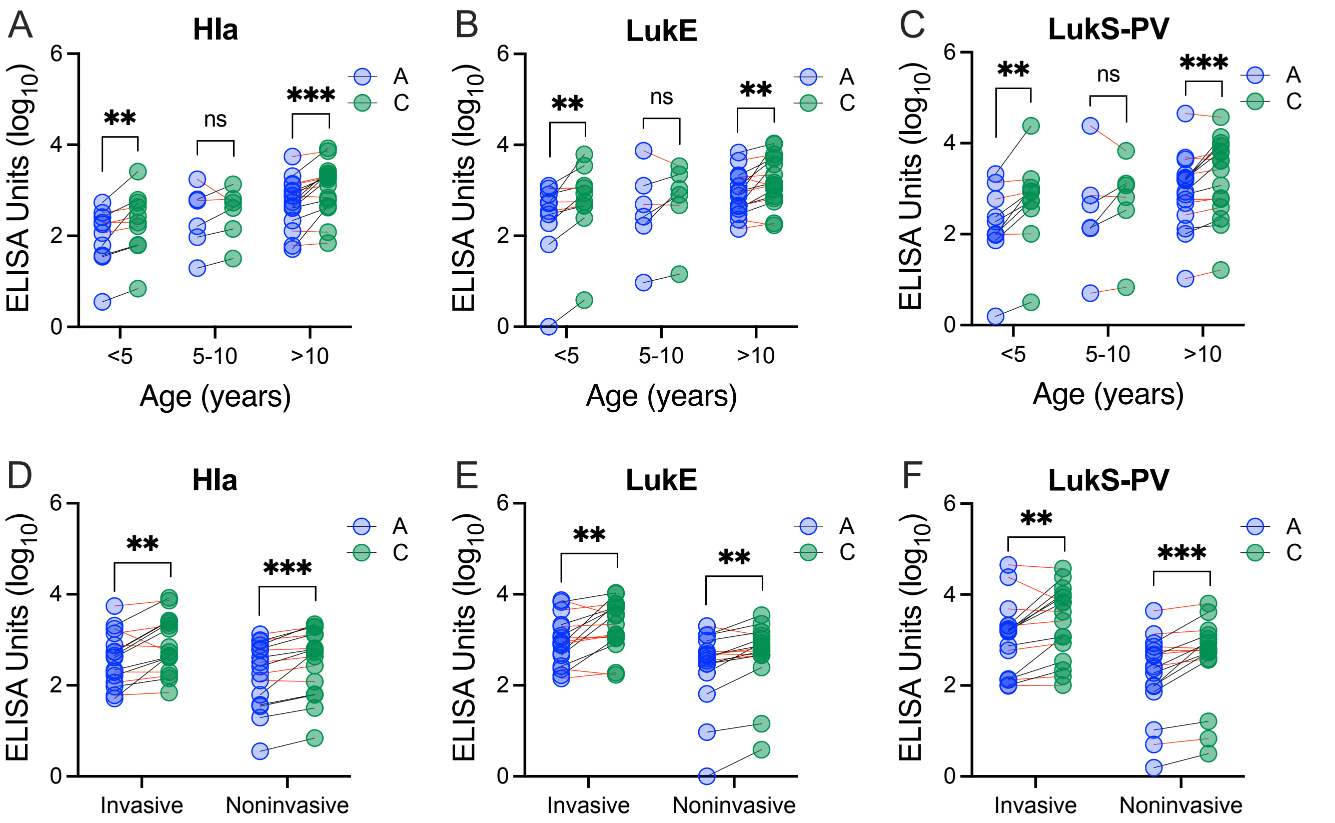

Supplementary Figure 1. Impact of age and severity of infection on infection-elicited increase in antibody levels. (A-C) Change in IgG levels against Hla (A), LukE (B), and LukS-PV (C) from acute *S. aureus* infection (A) to convalescence (C) based on the age of the study subject. (D-F) Change in IgG levels from acute infection to convalescence based on the severity of infection (invasive vs. non-invasive). Data are expressed as arbitrary ELISA units (log$_{10}$). Log$_{10}$-transformed values were compared by Wilcoxon matched-pairs signed rank test (A vs. C) or Mann-Whitney U test (P vs. NP). * indicates p<0.05; ** p<0.01; *** p<0.001; **** p<0.0001; ns not significant.

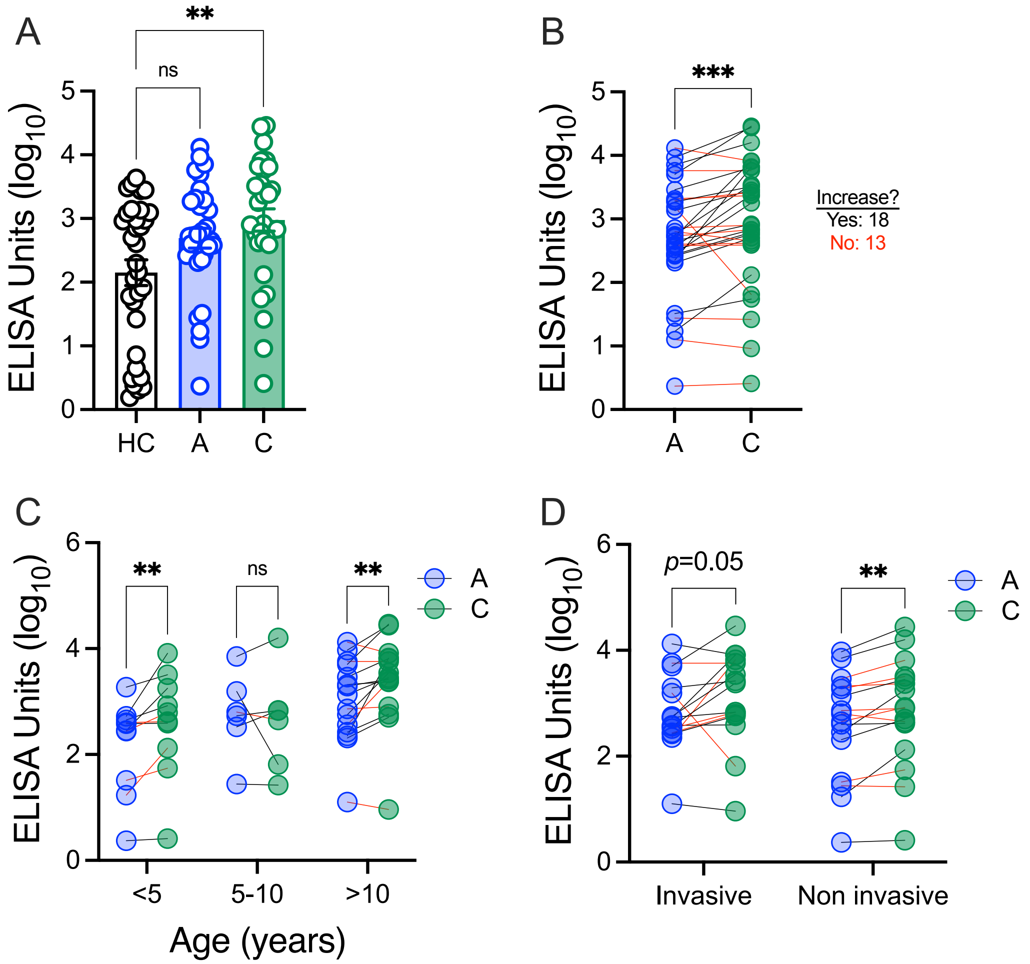

Supplementary Figure 2. Hla-specific IgG1 levels during acute infection and convalescence. (A) Levels of Hla-specific IgG1 in healthy children (HC), during acute *S. aureus* infection (A), or during convalescence (C). (B) Change in Hla-specific IgG1 levels for individual study subjects from acute infection to convalescence. (C) Change in IgG1 levels from acute *S. aureus* infection (A) to convalescence (C) based on the age of the study subject. (D) Change in IgG1 levels from acute infection to convalescence based on the severity of infection (invasive vs. non-invasive). Black lines indicate subjects for whom IgG1 levels increased ≥ 1.5 fold; red lines indicates subjects for whom IgG levels did not increase. Data are expressed as arbitrary ELISA units (log_{10}) and presented as the median values with individual values superimposed on the plots. Log_{10}-transformed values were compared by one-way ANOVA with Kruskal-Wallis post-test (A) or Wilcoxon matched-pairs signed rank test (B-D). * indicates *p*<0.05; ** *p*<0.01; *** *p*<0.001; ns not significant.

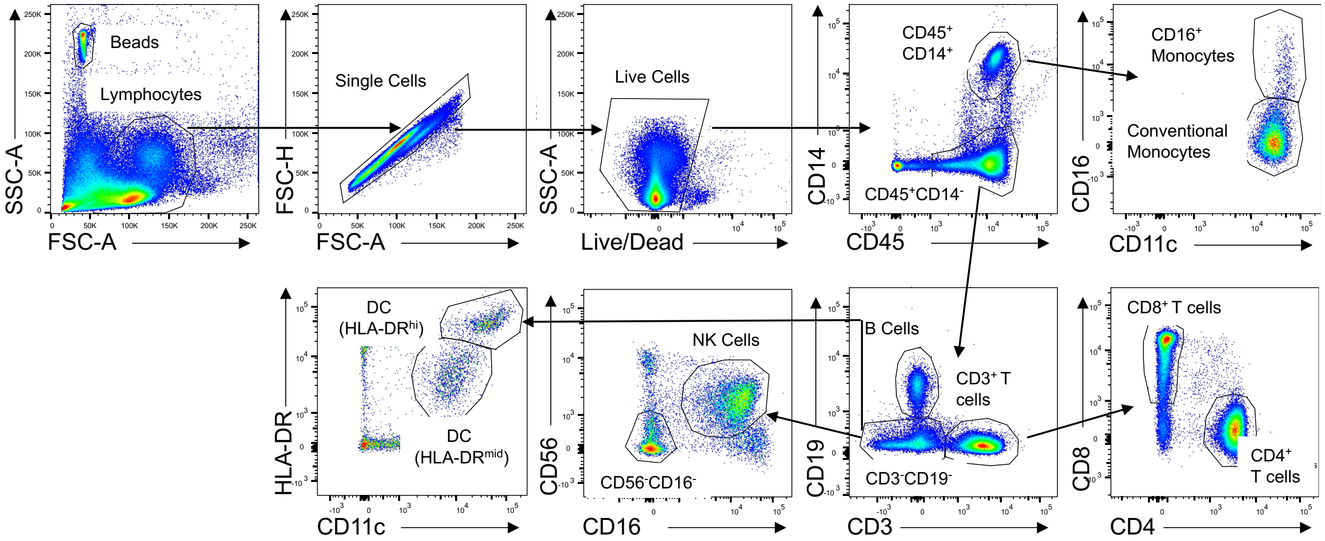

Supplementary Figure 3. Gating strategy for flow cytometric identification of immune cells. Cells were gated as single cells, followed by live/dead staining. Live cells were then gated as CD45$^+$CD14$^+$CD16$^-$ monocytes, CD45$^+$CD14$^-$CD3$^+$CD4$^{+/-}$CD8$^{+/-}$ T cells, CD45$^+$CD14$^-$CD3$^-$CD19$^+$ B cells, CD45$^+$CD14$^-$CD16$^+$CD56$^+$ NK cells, or CD45$^+$CD14$^-$CD11c$^+$HLA-DR^{hi} dendritic cells (DC).

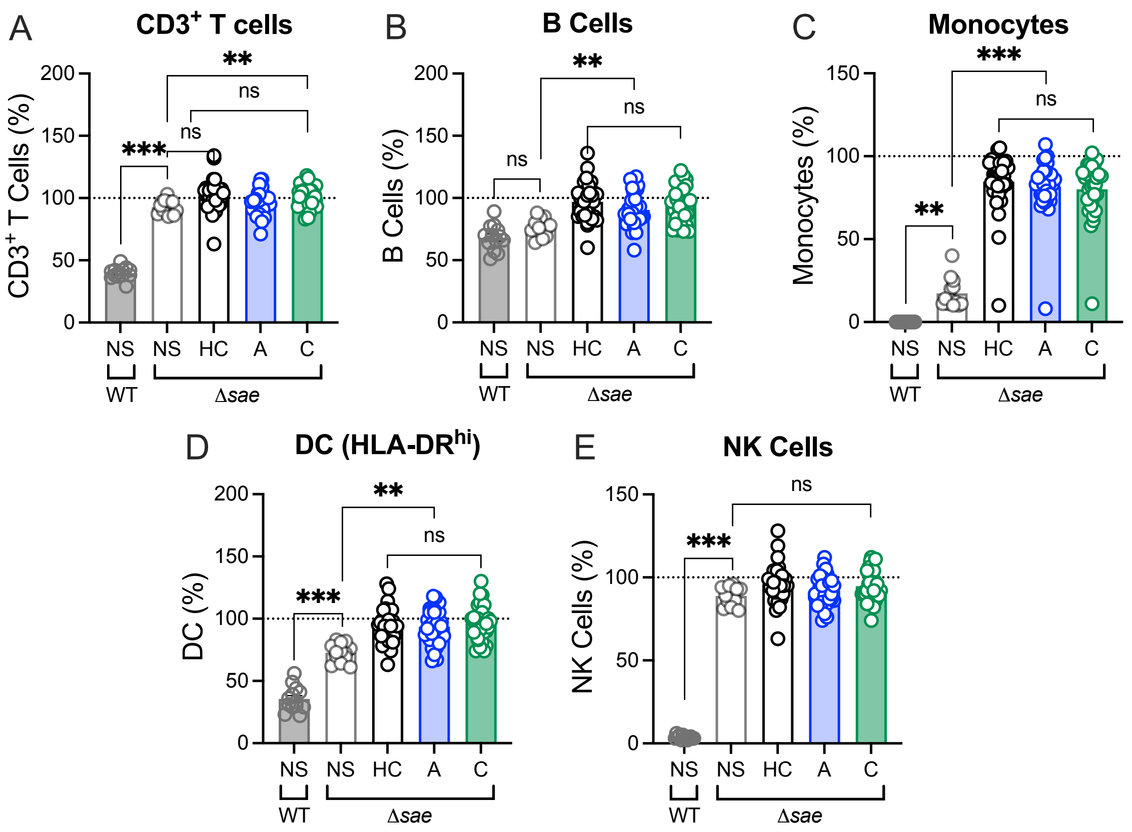

Supplementary Figure 4. Killing of immune cells is dependent on expression of *saeRS*-regulated toxins. PBMCs from healthy adults were incubated with wild-type (WT) or *Δsae S. aureus* supernatant and serum from study subjects (HC – healthy control; A – acute infection; C – convalescence) followed by quantification of live immune cells by flow cytometry (gating strategy, Fig S2). Compared with WT supernatant, that resulted in death of large numbers of T cells (A), monocytes (C), DC (D), and NK cells (E) in the absence of serum (NS), *Δsae* supernatant resulted in less killing (WT NS vs. *Δsae* NS). *Δsae* supernatant killed large numbers of monocytes (C), but neither WT nor *Δsae* supernatant was highly toxic to B cells (B). There were no significant differences in cell survival following incubation with *Δsae* among healthy children, acutely-infected children, or during convalescence. Data are expressed as % cell survival, compared with no supernatant control (dashed line at 100%) and presented as the median values with individual values superimposed on the plots. Data were compared by one-way ANOVA with Kruskal-Wallis post-test. * indicates p<0.05; ** p<0.01; *** p<0.001; **** p<0.0001; ns not significant.

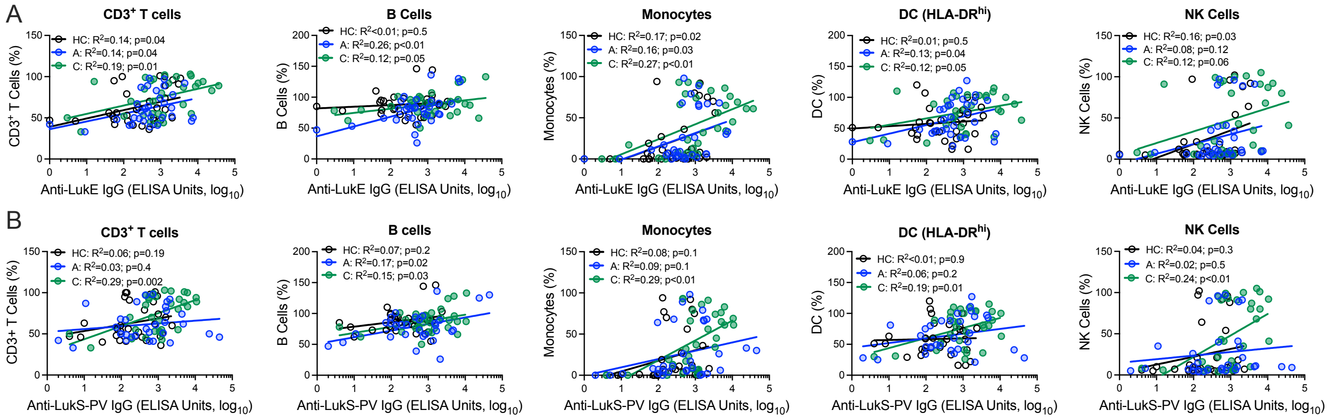

Supplementary Figure 5. Correlation of LukE- and LukS-PV-specific antibody levels with protection of immune cells against toxin-mediated killing. Correlation of LukE- (A) and LukS-PV-specific (B) IgG levels (ELISA units, log_{10}) with protection of T cells, B cells, Monocytes, Dendritic cells, and NK cells for healthy children (HC), acutely-infected children (A), or during convalescence (C). Correlations were determined by linear correlation using log_{10}-transformed IgG levels.

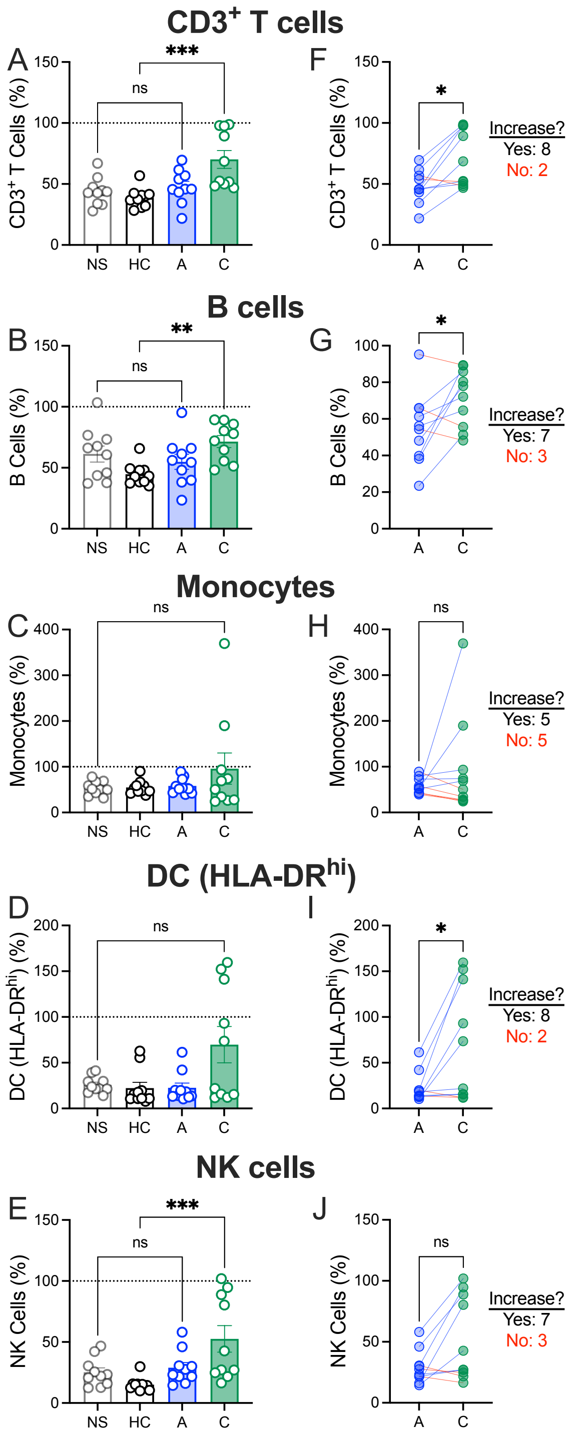

Supplementary Figure 6. Protection of immune cells against Hla-mediated killing by acute and convalescent sera. PBMCs from healthy adults were incubated with recombinant active Hla and serum from study subjects followed by quantification of live immune cells by flow cytometry (gating strategy, Fig S2). (A-E) Protection of CD3^{+} T cells (A), B cells (B), monocytes (C), HLA-DR^{hi} dendritic cells (DC)(D), and NK cells (E) by serum from healthy

children (HC), acutely-infected children (A), and during convalescence (C). “NS” indicates no serum controls. (F-J) Change in immune cell protection for individual study subjects from acute infection to convalescence. Black lines indicate subjects for whom immune cell survival increased ≥ 10%; red lines indicate subjects for whom cell survival did not increase. Data are expressed as % immune cell survival, compared with no supernatant control (not shown) and presented as the median values with individual values superimposed on the plots. Data were compared by one-way ANOVA with Kruskal-Wallis post-test (A-E) or Wilcoxon matched-pairs signed rank test (F-J). * indicates $p<0.05$; ** $p<0.01$; *** $p<0.001$; **** $p<0.0001$; ns not significant.

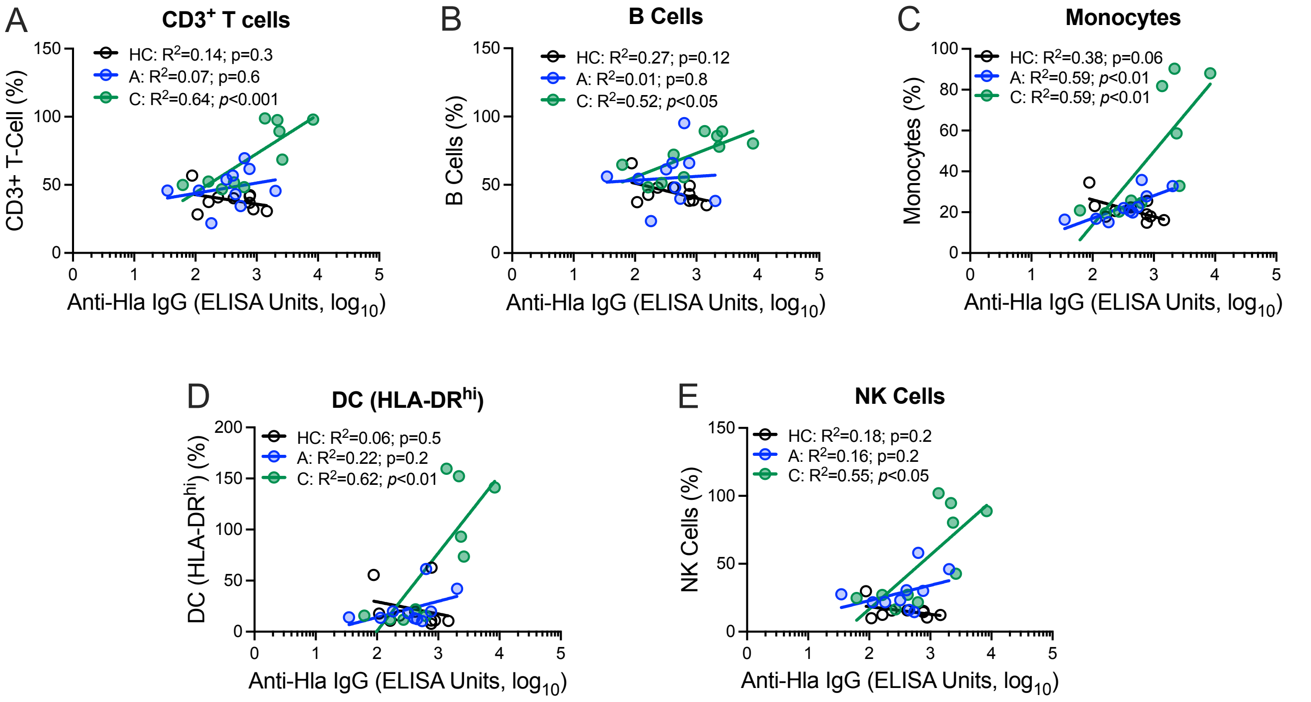

Supplementary Figure 7. Hla-specific IgG levels correlate with protection of immune cells against Hla-mediated killing. Correlation of Hla-specific IgG levels (ELISA units, $\log_{10}$) with protection of T cells (A), B cells (B), Monocytes (C), Dendritic cells (D), and NK cells (E) for healthy children (HC), acutely-infected children (A), or during convalescence (C). Correlations were determined by linear correlation using $\log_{10}$-transformed IgG levels.

Supplementary Table 1. Characteristics of children whose serum protects or does not protect immune cells against toxin-mediated killing.

		Not Protected	Protected	*p* value
Subjects		11	21	
Age in years Median (IQR)		5.8 (1.9, 9.0)	13.8 (8.3, 15.0)	0.047
Age Group				0.024
	<=5 yo	5 (45%)	5 (24%)	
	5-10 yo	4 (36%)	2 (9.5%)	
	>10 yo	2 (18%)	14 (67%)	
Gender				0.7
	Male	8 (73%)	17 (81%)	
	Female	3 (27%)	4 (19%)	
Race/Ethnicity				0.037
	White	7 (64%)	20 (95%)	
	Black	4 (36%)	1 (4.8%)	
Invasive Infection				>0.9
	Yes	6 (55%)	10 (48%)	
	No	5 (45%)	11 (52%)	
Susceptibility				>0.9
	MRSA	4 (36%)	7 (33%)	
	MSSA	7 (64%)	13 (62%)	
	Both	0 (0.0%)	1 (5%)	
Fever		7 (64%)	12 (57%)	>0.9
